# Is Edema Zone Volume Associated With Ki-67 Index in Glioblastoma Patients?

**DOI:** 10.7759/cureus.24246

**Published:** 2022-04-18

**Authors:** Ricardo Caramanti, Raysa M Aprígio, Carlos E D`Aglio Rocha, Dionei F Morais, Mário J Góes, Feres Chaddad-Neto, Waldir A Tognola

**Affiliations:** 1 Neurosurgery, Universidade Federal de São Paulo, São Paulo, BRA; 2 Neurosurgery, Hospital de Base de São José do Rio Preto, São José do Rio Preto, BRA; 3 Neurosurgery, Faculdade de Medicina de São José do Rio Preto, São José do Rio Preto, BRA; 4 Neurological Surgery, Universidade Federal De Sao Paulo-Unifesp, Sao Paulo, BRA; 5 Neurology, Faculdade de Medicina de São José do Rio Preto, São José do Rio Preto, BRA

**Keywords:** glioma, edema zone, ki-67 labeling index, volumetric analysis, glioblastoma

## Abstract

Introduction

Despite all the progress with genetic mapping and multimodal treatment, the prognosis of Glioblastoma multiforme (GBM) remains poor, with median overall survival (OS) of only 12 to 15 months. Several studies showed correlations between glioblastoma clinic and prognosis factors; however, it doesn`t occur with tumor radiological features. The purpose of this study is to determine possible correlations between the volumetric analysis of glioblastoma compartments and the proliferation index represented by Ki-67.

Methods

We performed a retrospective analysis of MRI studies of 70 patients with glioblastoma multiforme acquired up to one week before surgery. The tumor compartments were divided into enhancing zone; edema zone and tumor total zone. Each compartment was submitted to volumetric analysis using Horos Project software. A linear regression model was used to assess correlations between the ki-67 index and the volume of each compartment with a p-value of 0.05.

Results

The male/female ratio in our study was 1.7:1, at a mean age of 60.7 ± 14.6 years. Tumor predominant location was the temporal lobe with 25% of cases and cystic morphology was present in 17%. The median of Ki-67 was 40%. The average tumor compartment volume was 40 cm^3^ for the contrast-enhancing zone, 62 cm^3^for the edema zone, and 103 cm^3^ for the total tumor volume. A significant association between the Ki-67 index and edema zone volume (p=0.02) was found.

Conclusion

Volumetric analysis of the glioblastoma edema zone by MRI allows for predicting tumor aggressiveness through correlation with the Ki-67 index.

## Introduction

Glioblastoma multiforme (GBM) represents up to 81% of all primary malignant tumors of the central nervous system (CNS) [1]. This aggressive brain tumor has an incidence of four thousand new cases per year in the United States and increasing rates in Australia, Finland, South America, and Europe [1-4]. Despite all the progress with genetic mapping and multimodal treatment over the last decade, the prognosis of GBM remains poor, with median overall survival (OS) inferior of two years [5,6]. 

Established clinical variables used to predict patient prognosis include age, Karnofsky performance status (KPS), the extent of contrast-enhancing zone resection, and methylation status of O-6-methylguanine-DNA methyltransferase and chemo-radiotherapy protocol [7-10]. A recent meta-analysis showed an association between the overexpression of proliferation index Ki-67 and worse outcome scores [11].

On the other hand, a few investigators have assessed direct or indirect correlations between GBM compartment volume in magnetic resonance imaging (MRI) and prognosis. However, these findings have not been consistent so far [12,13]. The purpose of the present study was to determine possible correlations between the volumetric analysis of glioblastoma compartments and the proliferation index represented by Ki-67.

## Materials and methods

We performed a retrospective analysis of patients' records and magnetic resonance imaging (MRI) studies of 70 patients with glioblastoma multiforme that underwent surgical resection or stereotactic biopsy performed by three neurosurgeons in a single institution during the period between January 2014 and December 2018.

**Figure 1:**
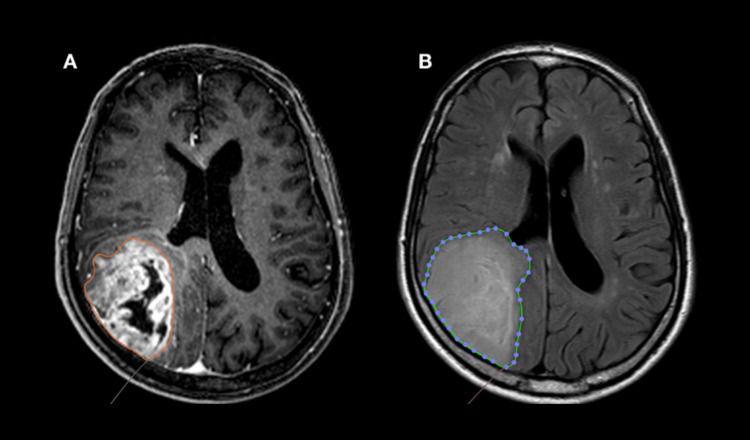
Glioblastoma multiforme MRI volumetric analysis a- MRI in T1-weighted sequence with gadolinium showing the calculation of lesion volume for enhancing zone. b - MRI in FLAIR-weighted sequence showing the calculation of lesion volume for tumor edema zone. Volumetric calculations were estimated with a manual tracer of the Horos software, by measuring the area of interest with a slice-by-slice semi-automatic method

Two pathologists confirmed tumor diagnosis through histological and immunohistochemical studies. All patient MRI scans were acquired up to one week before the surgical procedure with 1.5T or 3.0T machines. The midline shift (MS) was measured in millimeters by axial FLAIR sequence using the following equation: MS = (a/2) - b where “a” is the bihemispheric distance passing by the foramen of Monro and “b” is the distance from the pellucid septum to convexity in the deviated side. The volumetric measurement used the semi-automatic method by Horos Project software [14]. It was expressed in cubic centimeters (Figure 1).

MRI scans were done before any corticosteroids were used. Glioblastoma compartments were defined as: 

· Enhancing zone - Heterogeneous gadolinium-enhancing zone, measured by T1-contrast MRI sequence. 

· Edema zone - Zone of hyperintensity in FLAIR MRI sequence, adjacent to enhancing zone. 

· Total tumor zone - The sum of the volumes of enhancing and edema zones.

The statistical analyses were made using Minitab student release 14: Statistical software for education (Boston: Pearson Addison-Wesley) being applied descriptive statistics for categorical and continuous variables. We used a linear regression model to assess correlations between continuous variables. A p-value of 0.05 or lower was regarded as statistically significant.

This article was previously posted to the research square preprint server on May 28, 2021 [15]. This study was approved by the Fundação Faculdade Regional de Medicina de São José do Rio Preto (FAMERP) ethics committee and was performed according to institutional guidelines. The need for informed consent was waived by Fundação Faculdade Regional de Medicina de São José do Rio Preto (FAMERP) ethics committee for the present study.

## Results

The male/female ratio in our study was 1.7:1, at a mean age of 60.7 ± 14.6 years. Approximately 75% of patients were 60 years old or younger. Mean Karnofsky performance status was 83 ± 7.3% at patient admission. 

The predominant tumor location was the temporal lobe with 25% of cases, followed by frontal and parietal lobes with 21% each, occipital lobe with 20%, central core with 11%, and posterior fossa with 2% of cases. As for hemisphere distribution, 50% of tumors were in the left hemisphere, 47% were in the right hemisphere, and 3% of patients presented bilateral tumors. Lesions with cystic morphology were 17%. 

The most frequent clinical manifestations were motor deficits (22%), headache (20%), sensibility deficit (16%), seizures (15%), mental confusion (12%), speech altercations (12%), and visual deficit (3%) (Table 1). 

**Table 1:** Glioblastoma symptoms

Presentation	N	%
Headache	32	20
Seizure	22	15
Motor Deficit	33	22
Sensitive Deficit	24	16
Speech alterations	18	12
Visual Deficit	6	3
Confusion	19	12
Total	154	100
N= Number of occurrences		

The median of Ki-67 was 40%, and it did not appear to be related to age or sex. Midline shift was presented in 27 cases (38%) with an average of 7,5 millimeters. It had a significant statistical association with a motor deficit (p=0,03).

**Figure 2:**
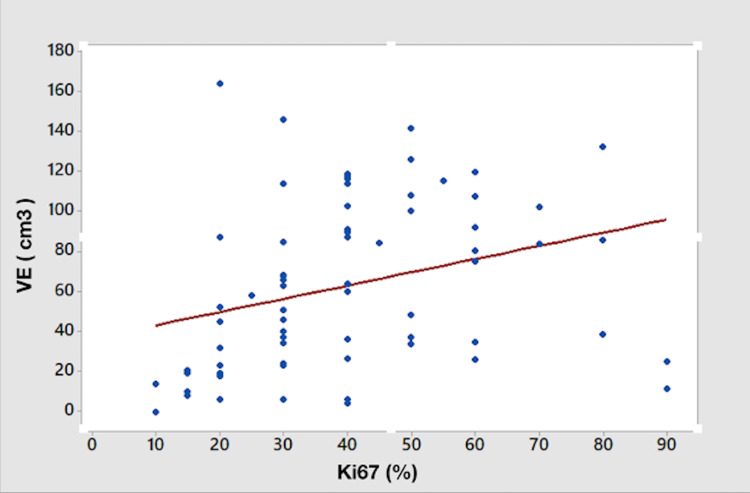
Scatter diagram showing positive correlation between Ki-67 index and volume of edema (p=0.02)

The median of Ki-67 was 40%, and it did not appear to be related to age or sex. The average tumor compartment volume was 40.8 cm3 for the contrast-enhancing zone, 62.7 cm3 for the edema zone, and 103.5 cm3 for total tumor volume. There was a significant association between Ki-67 index and edema zone volume (p=0.02), which was not the case for enhancing zone volume (p=0.10) and total tumor volume (p=0.33), (Figure 2).

## Discussion

Ki-67 is a protein associated with cell proliferation which increases during the mitotic process, mainly in the S phase of the cycle. The expression of Ki-67 in tumor cells, quantified in percentage, is called the Ki-67 index, which could be associated with prognosis in tumors such as melanomas, and breast and bladder carcinomas [16-19]. 

In 2000, Shimizu et al. [20] work findings revealed a relevant correlation between the Ki-67 index and choline levels on MR spectroscopy images. However, their research had limitations, such as a small number of patients, image artifacts, and possible errors in spectral evaluation [20,21]. 

Some authors support the idea of an association between the Ki-67 index and prognosis. Armocida et al. [22] showed that a Ki-67 index >20% was associated with shorter progression-free survival, which corroborates with Liang et al. [23] study that observed a correlation between the Ki-67 index and first-year mortality in 335 glioma patients. In their meta-analysis, Chen et al. [11] revealed that elevated levels of Ki-67 can be a predictive factor for poor prognosis in GBM.

Henker et al. [22] tried to show associations between tumor compartments volume and Ki-67 index in a study with 150 GBM patients, using 3D neuronavigation software; however, without success, probably due to inconsistent measurement methodology and software used. 

Armocida et al. [22] presented an association between high Ki-67 index and total volumes (>45 cm3) but not edema volumes, unlike our study. In this case, despite the use of similar measurement methods and software, compartment definition, which is not clearly stated, could differ from ours. Another divergence between findings of different studies is the proliferative index variation according to the site of extraction for biopsy, as proposed by Jakovlevs et al. [24].

The method for volumetric calculations in this study was quantitative and semi-automatic, which offers the advantage of manually demarcating the contours of each type of volume selected in each of the regions of interest (ROI) of an image. This allowed us to avoid deviations in lesion volume that commonly occur when a completely automatic methodology is applied. [25-27] 

Our study revealed a positive association between the Ki-67 index and edema zone volume in GBM. To the best of our knowledge, this is the first study to find this statistical correlation. The volume of enhancing zone and total tumor volume showed no association with a high proliferation index, as shown by other authors [21,25,26]. 

Despite these findings, a limitation of our research is the single-center retrospective format and, therefore, prospective studies are necessary to clarify the relation between glioblastoma compartment volume and clinical variables such as prognosis.

## Conclusions

Volumetric analysis of the glioblastoma edema zone by MRI shows a marginally significant increase in Ki-67 as edema volume increases, which should predict tumor aggressiveness and patient prognosis before biopsy realization and create a possibility of an early treatment plan.

Future prospective multicentric studies based on immunohistochemical, clinical, and radiological aspects of tumors are necessary to clarify this relationship and generate more elements to predict prognosis in GBM patients.
